# Network Connectance Analysis as a Tool to Understand Homeostasis of Plants under Environmental Changes

**DOI:** 10.3390/plants2030473

**Published:** 2013-07-10

**Authors:** Suzana C. Bertolli, Hilton F. Vítolo, Gustavo M. Souza

**Affiliations:** 1 Plant Ecological Cognition Laboratory, Western São Paulo University (UNOESTE), Rod. Raposo Tavares, km 572, 19067-175 Presidente Prudente, São Paulo, Brazil; E-Mail: fabriciovitolobio@gmail.com; 2 Department of Botany, São Paulo State University “Júlio de Mesquita Filho” (UNESP), Av. 24A, 1515, 13506-900 Rio Claro, São Paulo, Brazil; E-Mail: scbertolli@hotmail.com

**Keywords:** network connectance, physiological network, stability, water deficit, low and high temperature, C_3_ and C_4_ photosynthetic type metabolisms

## Abstract

The homeostasis of plants under environmental constraints may be maintained by alterations in the organization of their physiological networks. The ability to control a network depends on the strength of the connections between network elements, which is called network connectance. Herein, we intend to provide more evidence on the existence of a modulation pattern of photosynthetic networks, in response to adverse environmental conditions. Two species (*Glycine max*-C3 metabolism, and *Brachiaria brizantha*-C4 metabolism) were submitted to two environmental constraints (water availability, and high and low temperatures), and from the physiological parameters measured, the global connectance (*Cg_total_*) and the modules connectance (gas exchange-Cg_ge_ and photochemical-Cg_pho_) were analyzed. Both types of environmental constraints impaired the photosynthetic capacity and the growth of the plants, indicating loss of their homeostasis, but in different ways. The results showed that in general the *Cg_total_* of both species increased with temperature increment and water deficit, indicating a higher modulation of photosynthetic networks. However, the *Cg* variation in both species did not influence the total dry biomass that was reduced by environmental adversities. This outcome is probably associated with a loss of system homeostasis. The connectance network analyses indicated a possible lack of correspondence between the photosynthetic networks modulation patterns and the homeostasis loss. However, this kind of analysis can be a powerful tool to access the degree of stability of a biological system, as well as to allow greater understanding of the dynamics underlying the photosynthetic processes that maintain the identity of the systems under environmental adversities.

## 1. Introduction

Plant ecophysiology can be considered, *par excellence*, a science of the interactions between plant and environment. These interactions evolve direct and indirect responses, and feedbacks among diverse components of the plant in response to environmental changes, resulting in a complex network of interactions among elements of the system. Thus, approaches that assess and quantify systemic interactions, considering the relationships among the components of a system, could play an important role in improving classical ecophysiological methods of data analysis [1,2]. In addition, this is the kind of information that would have an important positive impact over the (i) predictions of vegetation behavior in face of climatic change scenarios, (ii) benefiting plant breeding programs, and (iii) the settlement of polices aiming to protect the most vulnerable sectors of agriculture and biodiversity hotpots, since it is possible to accurately define a system’s stability.

Although the first attempt to formalize a General Systems Theory had been held in the decades of 1950s–1960s by the theoretical bitologist Ludwig von Bertallanfy [3], only recently has this theory been applied to biology, especially molecular biology [4,5]. In plant biology, the focus of the Systems Theory approach has been applied to study the complex networks of interactions of the molecular signaling processes [6,7]. In this context, a huge number of studies were performed in order to describe large networks of gene regulation and protein interaction [8]. In fact, until now little attention has been given to the fundamental aspects regarding the organization of biological systems, trying to verify the capacity that these systems have to function under a wide range of environmental conditions within a certain degree of robustness [8]. Traditionally, this characteristic can be identified as the “homeostasis” of the system [9].

The Israeli researcher, G. Nissin Amzallag, proposed that the classical statistical approaches (based on mean comparison tests) were inappropriate to account for the complexity in the physiological patterns that underlie the maintenance of the homeostasis of plants in different phenological stages and under different environmental stimuli [10]. Amzallag has suggested, and also has provided, some experimental evidence of the maintenance of the homeostasis of plants by alterations in the organization of their functional networks. These changes in the networks allow the system to reach a steady state, resulting in an adjustment or in new patterns of connections among system’s elements, causing the maintenance of plant physiological characteristics when environmental adversities are occurring [10]. 

The susceptibility of physiological networks to environmental constraints depends on the strength with which their elements are connected, and also on the degree of linkage between the networks and the surrounding environment [2,11]. The strength of the connections between the elements of a network is called network connectance [10,12]. Elements strongly connected to each other (with high connectance) could promote a higher control ability to the network, since this characteristic of the network would enable the system to implement adjustments quickly and accurately because the responses would propagate faster through the network [9,13]. In general, the biological systems exhibit the ability to modulate their networks, *i.e*., they may change among different patterns of connectance according to the context, enabling the maintenance in the capacity of growth, protection, and reproduction under a wide range of environmental conditions [2,13].

The network analysis approach has been applied in plant physiological studies with cultivated species [1,9,14,15,16] and ecophysiological studies of tropical trees [2,11,17]. In general, such an approach is based on correlations among the physiological parameters, is very simple, and consumes little time. The results obtained by using the network analysis have provided important evidence about emerging patterns of plant responses to environmental constraints, contributing significantly to enlarging the understanding about the regulation of physiological processes, as well as to provide new parameters for plant breeding, specially seeking to incorporate resistance/tolerance characteristics to adverse environmental conditions. However the network regulation of important physiological processes such as photosynthesis, as well as its homeostasis in adverse environmental conditions, needs to be better elucidated in order to give a solid information basis to plant scientists, agronomists, and policy makers to develop their work.

In this context, it is worth mentioning that the use of network analysis has been particularly important when classical statistical approaches could not detect significant differences among treatments [10,16]. In the case of plant physiological studies, the absence of differences implies that the system under analysis was able to maintain its homeostasis in face of the diverse conditions. However, the maintenance of the homeostatic capacity does not indicate that the organisms have not been affected at all. Rather, the homeostasis maintenance of biological systems is based on a series of internal adjustments that allow the system to keep functioning within its own dynamic equilibrium [9,10].

By performing experiments with plants of different photosynthetic types of metabolism (C_3_ and C_4_) submitted to limiting conditions of temperature and water availability, we intend to provide more evidence on the presence of patterns of the photosynthetic networks modulation in response to environmental adversities, as well as to discuss new perspectives for the use of the systemic approach in plant ecophysiological studies, aiming to provide information about the stability of plant systems to environmental changes in a semi-quantitative and more effective way.

## 2. Results and Discussion

### 2.1. Physiological Responses to Environmental Constraints

The overall responses to environmental constraints followed the standard physiological patterns of plants exposed to water deficit [18,19] and temperature ranges [20,21,22].

In general, increments in air temperature increased photosynthetic capacity of both species, as indicated by higher values of *A_maxL_* at 40 °C (*p* < 0.05) (Table 1). On the other hand, the total respiration (dark respiration, *Rd* plus photorespiration, *Pr*) increased by 42% (39% in *Pr* and 49% in *Rd*) in *G. max*, but remained constant in *B. brizantha*, as temperature increased. There was an increasing trend of *A_maxCO2_* values under 40 °C, compared to a temperature of 30 °C, followed by the same trend in *V_cmax_* values. On the other hand, under 20 °C there was a limitation on biochemical activity of photosynthesis decreasing *A_maxCO2_* values (*p* < 0.05). 

**Table 1:** Mean values of photosynthetic capacity (*A_maxL_*), dark respiration (*R_d_*), photorespiration (*Pr*), photosynthetic potential (*A_maxCO2_*), relative stomatal limitation to photosynthesis (*Ls*), and maximum ratio of Rubisco carboxylation (*V_cmax_*) in *G. max* and *B. brizantha* under different temperature conditions (°C).

Gas exchange parameters						
	*G. max*			*B. brizantha*		
	20 °C	30 °C	40 °C	20 °C	30 °C	40 °C
*A_maxL_* *(µmol CO_2_ m^−2^ s^−1^)*	15.2 ^ab^	13.2 ^b^	18.5 ^a^	13.7 ^c^	20.3 ^b^	27.6 ^a^
*R_d_* *(µmol CO_2_ m−2 s^−1^*)	2.5 ^a^	1.2 ^b^	2.4 ^a^	2.1 ^a^	2.2 ^a^	2.4 ^a^
*P_r_* *(µmol CO_2_ m−2 s^−1^*)	3.2 ^b^	3.7 ^b^	6.1 ^a^	0.4 ^a^	0.3 ^a^	0.3 ^a^
*A_maxCO2_* *(µmol CO_2_ m−2 s^−1^)*	16.7 ^b^	20.2 ^b^	29.5 ^a^	11.1 ^b^	20.6 ^a^	25.4 ^a^
*L_S_*	9.8 ^b^	16.9 ^a^	20.9 ^a^	18.7 ^a^	14.6 ^a^	6.8 ^b^
V_cmax_ *(µmol m−2 s^−1^*)	61 ^b^	68 ^ab^	78 ^a^	18 ^a^	24 ^a^	27 ^a^

Different letters indicate statistical difference (*p* < 0.05) between temperature conditions to each species.

The results from chlorophyll fluorescence analysis, including alternative electron sink (*AES*), showed no significant damages over the photochemical machinery at 40 °C to both species. None of the analyzed photochemical parameters were affected by high temperature; instead of the apparent electron transport rate (*ETR*), which showed higher values at 40 °C for both species. On the other hand, low temperature significantly reduced photochemical efficiency, when compared to 30 °C, except for *ETR* in *G. max* (Table 2).

**Table 2:** Mean values of the potential (*F_v_/F_m_*) and effective *(∆F/F_m_’*) photosystem II quantum efficiency (PSII), the photochemical (*qP*), and the non-photochemical (*NPQ*) extinction coefficient, the apparent electron transport rate (*ETR*) and the alternative electron sink (*AES*) in *G. max* and *B. brizantha* under different temperature conditions (°C).

Photochemical parameters						
	*G. max*			*B. brizantha*		
	20 °C	30 °C	40 °C	20 °C	30 °C	40 °C
*F_v_/F_m_*	0.73 ^b^	0.74 ^b^	0.79 ^a^	0.75 ^b^	0.78 ^a^	0.79 ^a^
*ΔF/F_m_’*	0.18 ^c^	0.22 ^b^	0.25 ^a^	0.10 ^c^	0.15 ^b^	0.27 ^a^
*NPQ*	2.22 ^a^	2.20 ^a^	1.87 ^a^	2.78 ^a^	2.53 ^a^	1.81^b^
*ETR* *(µmol m^−2^ s^−1^)*	62 ^b^	57 ^b^	85 ^a^	42 ^c^	66 ^b^	94 ^a^
*AES*	6.2 ^a^	6.3 ^a^	7.1 ^a^	6.0 ^a^	4.1 ^a^	5.9 ^a^

Different letters indicate statistical difference (*p* < 0.05) between temperature conditions to each species.

In general, the mean values of the total dry mass (*DMt*) and leaf area (*LA*) were reduced in both species at temperatures of 20 and 40 °C, although the growth impairment was more expressive at 20 °C (Table 3). While growth reduction under 20 °C can be directly related to the low photosynthetic performance (Table 1), the lower plant growth under 40 °C, despite of the higher photosynthetic rates, was likely caused by an acceleration of senescence causing an impairment on plant growth rates [23,24].

**Table 3:** Mean values of total dry mass (*DMt*) and leaf area (*LA*) in *G. max* and *B. brizantha* under different temperature conditions (°C).

Growth parameters						
	*G. max*			*B. brizantha*		
	20 °C	30 °C	40 °C	20 °C	30 °C	40 °C
*DMt (g)*	5.90 ^c^	12.97 ^a^	9.67 ^b^	13.10 ^c^	32.74 ^a^	22.54 ^b^
*LA (m^2^)*	0.05 ^c^	0.10 ^a^	0.07 ^b^	0.09 ^c^	0.23 ^a^	0.13 ^b^

Different letters indicate statistical difference (*p* < 0.05) between temperature conditions to each species.

The imposed condition of water deficit decreased photosynthetic capacity in both species (Table 4). Our results also showed that, by the classical statistical approach, the homeostasis of the photochemical apparatus was maintained in both species (Table 5). Thus, the decrease in photosynthesis was probably caused by biochemical constraints, as supported by the significant reduction in *V_cmax_* with the decrease in water availability for both species. Concerning the growth parameters, it was observed that the water deficit condition significantly reduced the total dry mass, although the leaf area was maintained for plants of both species (Table 6).

**Table 4:** Mean values of photosynthetic capacity (*A_maxL_*), dark respiration (*R_d_*), photorespiration (*Pr*), photosynthetic potential (*A_maxCO2_*), relative stomatal limitation to photosynthesis (*L_S_*), and maximum ratio of Rubisco carboxylation (*V_cmax_*) in *G. max* and *B. brizantha* under different water regimes (100% and 30% refill of the total amount of the evapontranspired water).

Gas exchange parameters				
	*G. max*		*B. brizantha*	
	100%	30%	100%	30%
*A_maxL_* *(µmol CO_2_ m^−2^ s^−1^)*	17.3 ^a^	10.98 ^b^	14.07 ^a^	6.86 ^b^
*R_d_* *(µmol CO_2_ m^−2^ s^−1^ )*	2.28 ^a^	2.07 ^a^	1.23 ^a^	0.86 ^a^
*Pr* *(µmol CO_2_ m^−2^ s^−1^)*	5.25 ^a^	3.40 ^b^	0.07 ^a^	0.08 ^a^
*A_maxCO2_* *(µmol CO_2_ m^−2^ s^−1^)*	18.68 ^a^	6.80 ^b^	21.78 ^a^	10.46 ^b^
*L_S_*	27.9^a^	28.7 ^a^	9.6 ^b^	18.8 ^a^
*V_cmax_* *(µmol m^−2^ s^−1^)*	84 ^a^	61 ^b^	21 ^a^	10 ^b^

Different letters indicate statistical difference (*p* < 0.05) between water regimes to each species.

**Table 5:** Mean values of the potential (*F_v_/F_m_*) and effective *(∆F/F_m_’*) photosystem II (PSII) quantum efficiency, the photochemical (*qP*) and the non-photochemical (*NPQ*) extinction coefficient, and the apparent electron transport rate (*ETR*) in *G. max* and *B. brizantha* under different water regimes (100% and 30% refill of the total amount of the evapontranspired water).

Photochemical parameters				
	*G. max*		*B. brizantha*	
	100%	30%	100%	30%
*F_v_/F_m_*	0.788 ^a^	0.817 ^a^	0.783 ^a^	0.786 ^a^
*ΔF/F_m_’*	0.273 ^b^	0.404 ^a^	0.196 ^a^	0.208 ^a^
*NPQ*	2.07 ^a^	1.70 ^b^	3.15 ^a^	3.10 ^a^
*ETR* *(µmol m^−2^ s^−1^)*	89 ^a^	86 ^a^	64 ^a^	44 ^b^
*DAE*	10.7 ^b^	21.8 ^a^	8.8 ^a^	12.4 ^a^

Different letters indicate statistical difference (*p* < 0.05) between water regimes (100% or 30%) to each species.

**Table 6:** Mean values of total dry mass (*DMt*) and leaf area (*LA*) in *G. max* and *B. brizantha* under different water regimes (100% and 30% refill of the total amount of the evapontranspired water).

Growth parameters					
	*G. max*		*B. brizantha*		
	100%	30%	100%	30%	
*DMt (g)*	72.3 ^a^	19.7 ^b^	147.7 ^a^	48.3 ^b^	
*LA (m^2^)*	43 ^a^	41 ^a^	53 ^a^	49 ^a^	

Different letters indicate statistical difference (*p* < 0.05) between water regimes (100% or 30%) to each species.

The effects of water deficit on photosynthesis and plant growth have been widely studied and some mechanisms of plant response to this environmental adversity are well described in a great number of studies [18,19,25,26]. In general, water deficit disturbs the energy budget between the capture and the conversion metabolism of energy. Under moderate water availability, the photochemical processes are not significantly affected, while the biochemistry of CO_2_ fixation can be negatively impacted, reducing the carbon assimilation by the plant, affecting plant primary production. The causes of the reduced biochemical activity in this condition involve different factors. First of all, it is common to observe the stomatal limitation to CO_2_ diffusion to the carboxylation sites in the mesophyll. After, it is also possible that other effects take place, as a decrease in the mesophyll conductance, the limitation of the synthesis of ribulose bi-phosphate (RuBP), and even the inhibition of enzymes of the Calvin cycle.

Since the biochemical processes are deactivated for one of those reasons, the most important sink of photochemical energy ceases functioning, leading the photosynthetic machinery to a state of excess of energy. The exceeding energy not used in the CO_2_ assimilation process generates a surplus energy to be dissipated, being a problem to the plant that obligatorily has to deal with this extra energy, which can cause oxidative damages to the thylakoids membrane. In this situation an increase is observed in the NPQ, however this non-photochemical dissipation is often insufficient to remove the extra energy, resulting in the production of reactive oxygen species (ROS) that can damage the photosystems, particularly the D1 protein of PSII, and cause damage to ATP synthase. The decrease in the pool of ATP decreases the synthesis of RuBP, resulting in reduced potential photosynthesis accompanied by decreased activity of Rubisco [18,27,28]. Of course, the reduced carbon assimilation directly affected dry mass accumulation by the plants, reducing the overall plant growth even if the leaf area is maintained. We believe that this cascade effect could explain the response patterns of photosynthesis and growth to water availability observed in our results for both species. 

### 2.2. Network Modulation Facing Environmental Adversities

The results of network analysis, considering the variations in the modulation of photosynthetic networks, showed some differences between the two species concerning how the photosynthetic apparatus adjusts to environmental constraints.

Focusing on the results obtained by exposing individuals of both species to diverse air temperature conditions (Table 7), we observed a general increase trend in the *Cg_total_*, *Cg_ge_*, and *Cg_pho_* values as temperature increased. This trend was more expressive in *B. brizantha* (82% increase in *Cg_total_*). 

**Table 7:** Global connectance values of all physiological network (*Cg_total_*), of the photochemical network (*Cg_pho_*) and of the gas exchange network (*Cg_ge_*), and the relation between the photochemical module and the gas exchange module of the network, represented by the ETR-*A_maxL_* ratio observed in *G. max* and in *B. brizantha* under different temperatures.

	*G. max*			*B. brizantha*		
	20 °C	30 °C	40 °C	20 °C	30 °C	40 °C
*Cg_pho_*	1.68	1.82	2.42	1.29	0.88	2.01
*Cg_ge_*	1.22	2.31	2.65	1.27	1.35	2.08
*Cg_total_*	*1.45*	*2.06*	*2.54*	*1.29*	*1.12*	*2.04*
*ETR-A_maxL_*	0.769	0.668	0.987	0.591	0.822	0.953

In addition, it is worth to notice that under 20 °C, the *G. max* *Cg_total_* decreased by 29%, while in *B. brizantha* Cg_total_ increased by 15% in comparison with values observed at 30 °C. The lowest *Cg_total_* values observed in plants under 20 °C compared with plants cultivated at 40 °C are probably associated with the values of total dry mass, which were significantly higher in the 40 °C in relation to the 20 °C condition. These results can indicate that higher modulation (adjustment) in the photosynthetic networks might have contributed to a greater acclimation of plants of both species at 40 °C, improving biomass production. Again, this result could be more related to the decrease in the photosynthetic capacity at 20 °C than to the increase of total respiration observed at 40 °C. The total biomass in *G. max* and *B. brizantha* were reduced respectively by 54 and 60% at 20 °C, while the reduction under 40 °C was 25 and 31%, respectively, based on comparison of the total dry mass obtained at 30 °C. 

In addition, a generalized trend of increased linkage can be observed between the photochemical and gas exchange modules of the photosynthetic network, represented by the *ETR-A_maxL_* ratio, as temperature increases. This greater coupling among network modules should confer greater stability on the photochemical energy supply for maintaining the biochemical process of carbon assimilation. At 20 °C, this ratio was expressively lower in *B. brizantha* in relation to *G. max*, confirming that at the photosynthetic C_4_ type metabolism is more sensitive to low temperatures than C_3_. 

Considering that the number and the strength of connections among elements of a network are strongly related to the system stability [2,9,29,30,31], higher connectance values may indicate great system stability, up to a critical threshold [12]. In this case, our results showed solid evidences of the modulation of the photosynthetic networks, in C_3_ and C_4_ plants, in response to a temperature range. The studied systems changed their network patterns of connectance in order to arrive at a steady state. The higher the temperature was, the greater was the need of network adjustments to keep the system functioning. This is because tighter networks, with strong relationships among elements, could provide greater levels of system control, improving the system’s capacity to overcome external perturbations. Therefore, changes in system connectance may be considered as an adaptive response to environmental constraints [2,9].

Regarding the modulation of photosynthetic networks in response to water deficit (Table 8), *Cg_total_*, *Cg_ge_*, and *Cg_pho_* values increased with the reduction in the amount of water availability in both species. Moreover, unlike what was observed in the responses to different temperatures, the variations in the modulation of photosynthetic networks under water deficit did not exhibit a common response pattern to both species. For instance, the great differences in the *Cg_total_* values with the water regimes in both species did not result in major differences in the total dry mass gain with watering, which were similar to *G. max* (72%) and *B. brizantha* (66%). Changes in the photosynthetic capacity of plants (*A_maxL_*) were also weakly correlated with changes in *Cg*. These results suggest that changes in the patterns of modulation of photosynthetic networks in response to water deficit were not effective to maintain homeostasis of the studied plants, or even to promote a more appropriate physiological state for acclimation. These results differ significantly from what was reported in sugarcane plants [16], where higher *Cg* values of the photosynthetic networks contributed to the homeostasis maintenance of plants under severe water stress.

**Table 8:** Global connectance values of all physiological network (*Cg_total_*), of the photochemical network (*Cg_pho_*) and of the gas exchange network (*Cg_ge_*), and the relation between the photochemical module and the gas exchange module of the network, represented by the ETR-*A_maxL_* ratio observed in *G. max* and in *B. brizantha* under different water regimes (100% or 30%).

	*G. max*		*B. brizantha*	
	100%	30%	100%	30%
Cg_pho_	4.09	5.07	2.91	3.04
Cg_ge_	1.28	1.42	0.90	1.92
*Cg_total_*	*2.7*	*3.2*	*1.9*	*2.5*
*ETR-A_maxL_*	0.85	0.97	0.31	0.93

Moreover, the connection between the photochemical and the gas exchange modules showed a higher linkage between the modules in plants under water deficit. This response was particularly significant in *B. brizantha*, indicating a higher coupling between the biochemical and photochemical apparatus, in a more adverse environment, as also observed by [2].

### 2.3. Concerns about the Future Directions of Network Analysis of Photosynthesis

In a climate change scenario, concerns about the stability of the photosynthesis process is of particular importance, as this is the most important process in the global carbon biogeochemical cycle and because there are, yet, a lot of uncertainties regarding the behavior of this process in a warmer, drier, and more CO_2_ rich atmosphere [32]. Some tentative experimental approaches have been applied to predict the photosynthetic patterns of plants in those relatively new environmental conditions at the leaf level [33,34], little advance was done in the sense of quantifying the photosynthesis stability, as shown here. Bearing this in mind, and considering the experimental and analytical limitations of our experiments, it was possible to provide substantial evidences that the C_3_ and C_4_ species exhibit a high modulation capacity of the network processes related to carbon assimilation to high temperature and to water deficit, but that this modulation capacity was unlikely to promote the stability of the systems. However, the major outcome of our research was the possibility to verify these adjustment patterns by applying the systemic analysis approach. Other studies, performed in similar contexts, also had similar outcomes [2,9,16]. Nonetheless, one of our results pointed out that one limitation of the systemic approach, which still needs to be overcome in the short term, is how to adequately quantify photosynthesis network stability.

The first problem to be solved can be stated as the definition of what is stability of a biological system. The term stability is still undefined in the scientific community [35]. We assume that the stability of a biological system involves, in general, three basic properties: (1) homeostasis, defined as the tendency of internal adjustments of the organism aiming to maintain the basic processes and mechanisms constant even when facing changes in the external conditions [36], which confers the primary resistance of the system to the changes induced by the surrounding environment; (2) resilience, generally defined as the return capacity of the system to its initial state (normal) after an external perturbation [35,37], conferring flexibility to the system; and (3) persistence, which is the capacity of the system to maintain its identity as a whole [35].

In the new Science of System Biology, these concepts are considered in the framework of robustness [38]. Robustness is broadly defined as the ability of biological systems to keep functioning in the face of perturbations. Such perturbations can be induced by genetic mutations (internal changes) or by environmental constraints (external changes). However, a core issue related to the robustness of the system is: What feature of the system must be considered to determine its robustness? According to Wagner [38], ultimately, the fitness of the system would be the only feature that really matters. However, fitness is difficult to strictly define and even harder to measure. Therefore, to access the robustness or stability of a biological system one should consider its totality, including its different organizational levels [39,40,41]. In the case of plants, aspects of organization of photosynthetic networks are essential to be considered in stability analysis [9].

Another aspect is that biological systems are organized as networks with a variety of interconnection and hierarchical attributes [8,42,43]. The existence of significant redundancy within the network buffers, the primary pathways, or mechanisms within biological systems against external perturbations [30]. Systems with sufficient redundancy provide robustness in performance even when the system suffers an external disturbance, e.g., via transmission across alternate pathways, providing overall stability to the ensemble system [10,30]. Specifically, the quantity and the strength of the connections between network components have been directly correlated with the system stability [2,29,30]. The ability of the system to move among different states of organization provides the system with more stability. Wagner [38] calls this “neutral space”, which is the collection of solutions equivalent to the same biological problem. Thus, the more robust the system is, the larger the associated neutral space will be.

Moreover, network connectance analysis could provide valuable information to models of controllability of complex networks [44]. Network controllability is the ability to drive a system’s behavior towards a desired state through an adequate handling of some input parameters. In general, the models of controllability are based on the identification of sets of driver nodes that can guide the system’s dynamics [44]. On the other hand, Ferrarini [45] has also suggested to integrate the control of the edges (links) between the nodes as complementary to the models of network controllability. 

Thus, in this context, the understanding of how the strength of the links among the elements of the system changes in different environmental conditions could provide important information for further modeling of photosynthesis dynamics.

## 3. Experimental Section

### 3.1. Plant Material and Experimental Design

The crop species compared in this study were *Glycine max* (L.) Merrill cv. CD 202, a species with a photosynthetic C_3_ type metabolism, and *Brachiaria brizantha* cv. Marandú, a species with a photosynthetic C_4_ type metabolism. Both species are extensively cultivated in the tropical and subtropical regions of Brazil (southeast and central-west). The *G. max* and *B. brizantha* plants were seed germinated under greenhouse conditions in containers containing 12 kg of a 1:1 blend of red-yellow Ultisol soil with organic substratum, and watered daily. The growth conditions in both experiments were monitored daily by an automatic temperature and humidity measuring device (HOBO model H08-004-02, EUA), and the irradiance was evaluated with a quantum sensor (model Li-190SA), connected to a digital reader (model Li-250A, Li-Cor, EUA). 

#### 3.1.1. Temperature Conditions Experiment

Plants of both species were subjected to three day/night temperature conditions in a growth chamber (model EL 011, Eletrolab, SP, Brazil). Inside the growth chamber the relative air humidity was maintained at 60% under a 16/8 h (light/dark) photoperiod and a photosynthetic photon flux density (PPFD) of 600 μmol m^−2^ s^−1^. The temperature conditions were established by varying the diurnal temperature (20, 30, and 40 °C) and fixing the nocturnal temperature (20 °C). The diurnal temperatures were defined based on analysis of photosynthetic curves in response to temperature performed by infrared gas exchange measurements (LI-6400, Li-Cor, NE, USA). In this analysis, the sampling chamber temperature varied from 15 to 45 °C, with constant irradiance (1,200 μmol m^−2^ s^−1^), CO_2_ concentration (380 μmol mol^−1^), and humidity (60%) (data not shown). With *G. max*, plants were submitted to the temperature conditions after the fourth trifoliate leaflet was completely expanded (phenological stage V4). For *B. brizantha*, plants were cut 20 cm above surface soil level fifty days after germination in order to standardize plant size prior to treatment application. The plants were irrigated once a day at full field capacity and were grown for thirty days under the three temperature conditions. The study was carried out in a 2 × 3 factorial design (two species and three temperature conditions) with seven replications (plants).

#### 3.1.2. Water Deficit Experiment

Plants of both species were submitted to two water availability conditions: one with a 100% refill of the total amount of the evapontranspired water and another with a 30% refill of the total amount of the evapontranspired water, during thirty days, in green house conditions. The water replacement was performed by the gravimetric method, weighing the pots daily. The *G. max* and *B. brizantha* were submitted to the watering conditions in the growth chamber with the same environmental parameters way as described in the temperatures experiments (relative air humidity, photoperiod, and PPFD). The temperature inside growth chamber was 30/22 °C day/night. The plants were grown for thirty days under the watering conditions. The study was carried out in a 2 × 2 factorial design (two species and two watering conditions) with seven replications (plants).

### 3.2. Analyses of the Physiological Variables

Photosynthetic response curves to CO_2_ were performed in eight plants of each experimental condition (*A/Ci* curves, where A corresponds to the CO_2_ net assimilation and *Ci* to the intercellular CO_2_ concentration) as well as photosynthetic response curves to light (*A/PPFD*, where *PPFD* is the photosynthetic photons flux density), according to standard procedures described in [46,47], respectively. The *A/Ci* and the *A/PPFD* curves were adjusted using the fitting models proposed by [46,48]. All *A/Ci* and *A/PPFD* curves were performed using measurements taken from healthy fully expanded leaves from 9:00 a.m. to 1:00 p.m. The temperature of the IRGA leaf chamber (Li-6400XTR, LiCor, EUA) was adjusted to 30 °C and the vapour pressure deficit was maintained at 1.5 kPa with the aid of a dew point generator (model Li-610, Li-Cor) connected to the chamber. The light was provided by LEDs emitting in the blue-red spectrum, connected to the Li-6400XTR sampling chamber. The photosynthetic potential (*A_maxCO2_*) was obtained from *A/Ci* curves [46], and the photosynthetic capacity (*A_maxL_*), as well as dark respiration (*R_d_*), were obtained from *A/PPFD* curves [47]. The photorespiration (*Pr*) and maximum ratio of Rubisco carboxylation (*V_cmax_*) were calculated for C_3_ [49,50] and C_4_ [51] types photosynthetic metabolism. The relative stomatal limitation to photosynthesis (*L_S_*) was calculated [52]. 

To evaluate the photochemical apparatus, fluorescence analysis of chlorophyll a was carried out simultaneously with the *A/PPFD* curves, using a modulated light fluorometer (LI-6400-40) connected to the Li-6400XTR. The estimated parameters were the potential (*F_v_/F_m_*) and the effective *(∆F/F_m_*’) photosystem II (PSII) quantum efficiency, the non-photochemical (*NPQ*) of extinction coefficient and the apparent electron transport rate (*ETR*) [53,54,55]. The alternative electron sink (*AES*) was estimated according to [56].

### 3.3. Growth Analysis

The total leaf area was measured with a portable leaf area integrator (model LI-3000A, Li-Cor, Lincoln, NE, USA). To quantify the leaf dry mass, leaves were collected in paper bags and kept in a vented drier (70 °C) until constant weight. At the end of the experimental period, total plant dry mass was measured by drying the whole plants (above and below ground parts), as mentioned above. 

### 3.4. Data Analysis

The data were analyzed using the classical analysis of variance (ANOVA) approach, and the mean values were compared by the Tukey’s test (*p* < 0.05). 

To assess changes in the photosynthetic network connectance, we evaluated differences in modulation of gas exchange and chlorophyll fluorescence networks when individuals were submitted to the different experimental conditions. Modulation, here, was considered as the change in the mean strength of connections among network elements, which was measured through global connectance, *Cg*, following the concept and determination of Amzallag [10]. To define connectance, we specified a collection of paired variables of interest in the network. The correlation coefficients (r) between each paired variables were used to test the significance of the correlation and to measure the strength of the relationship, performing a z-transformation afterwards [10] where: z = 0.5 ln [(1 + |r|)/(1 − |r|)]. Global network connectance (*Cg*) was calculated as the average of z-values [10]. The photosynthetic process was separated into two representative networks constituted by pairs of parameters [2]. The leaf gas exchange network was composed by the following relationships: *A_maxL_-g_s_*, *A_maxL_-E*, *A_maxL_-R_d_*, *A_maxL_-Pr*, *A_maxL_-Ci*, *Ci-g_s_*, *Ci-R_d_*, *Ci-Pr*, and *g_s_-E*, where *A_maxL_* is the photosynthetic capacity, *g_s_* is the stomatal conductance, *E* is the transpiration rate, *R_d_* is the dark respiration, *Pr* is the photorespiration, and *Ci* is the internal CO_2_-concentration, and their average strength yields the global connectance of the gas exchange network (*Cg_ge_*). The photochemical network was formed by the relationships *∆F/F_m_’-ETR*, *∆F/F_m_’-F_v_/F_m_*, *∆F/F_m_*’*-NPQ*, *ETR-NPQ*, *F_v_/F_m_-NPQ*, and *F_v_/F_m_-ETR*, and their average strength yields the global connectance of the photochemical network (*Cg_pho_*). The *ETR-A* relationship linked the gas exchange and photochemical networks, and the average strength of both networks yields the photosynthetic network connectance (*Cg_total_*) [2].

## 4. Conclusions

Considering the great complexity of the photosynthetic process as a whole (gas exchanges/biochemical and photochemical processes), we believe that the use of global connectance analysis, associated with multi-scale analysis [41], can be a powerful tool to access the degree of physiological stability of a plant system, allowing the better understanding of the dynamics underlying the processes that maintain the identity of the systems under environmental constraints. 

On the other hand, it seems that the global connectance analysis is more appropriate to explain the causes of systemic homeostasis maintenance (when no major changes in the plant are caused by environmental disturbances) than to provide a causal explanatory basis to the loss of homeostasis. Rather, the application of analysis of *Cg* to our current data seems to indicate a possible lack of correspondence between the pattern of modulation of the networks and the break of the homeostasis of the system. In this way, new experimental results and/or meta-analysis, and the incorporations of concepts like robustness, resistance, and resilience to the analytical framework, are needed to further validation and to provide reliable interpretation of network analyses.
